# A Serious Game to Improve Emotion Regulation in Treatment-Seeking Individuals With Gambling Disorder: A Usability Study

**DOI:** 10.3389/fpsyg.2021.621953

**Published:** 2021-03-04

**Authors:** Teresa Mena-Moreno, Fernando Fernández-Aranda, Roser Granero, Lucero Munguía, Trevor Steward, Hibai López-González, Amparo del Pino-Gutiérrez, María Lozano-Madrid, Mónica Gómez-Peña, Laura Moragas, Isabelle Giroux, Marie Grall-Bronnec, Anne Sauvaget, Bernat Mora-Maltas, Eduardo Valenciano-Mendoza, José M. Menchón, Susana Jiménez-Murcia

**Affiliations:** ^1^Department of Psychiatry, University Hospital of Bellvitge-IDIBELL, Barcelona, Spain; ^2^Ciber Fisiopatología Obesidad y Nutrición (CIBERObn), Instituto de Salud Carlos III, Madrid, Spain; ^3^Department of Clinical Sciences, School of Medicine and Health Sciences, University of Barcelona, Barcelona, Spain; ^4^Department of Psychobiology and Methodology, Autonomous University of Barcelona, Barcelona, Spain; ^5^Melbourne School of Psychological Sciences, University of Melbourne, Parkville, VIC, Australia; ^6^Department of Public Health, Mental Health and Perinatal Nursing, School of Nursing, University of Barcelona, Barcelona, Spain; ^7^Centre d’Excellence pour la Prévention et le Traitement du Jeu, Faculté de Sciences Sociales, Université Laval, Pavillon Félix-Antoine-Savard, Quebec, QC, Canada; ^8^INSERM, SPHERE U1246, University of Nantes, Nantes, France; ^9^CHU Nantes, Movement ‐ Interactions ‐ Performance, MIP, University of Nantes, Nantes, France; ^10^Ciber Salut Mental (CIBERSAM), Instituto de Salud Carlos III, Madrid, Spain

**Keywords:** new technologies, serious games, heart rate variability, emotion regulation, gambling disorder

## Abstract

**Background**: Serious games have shown positive results in increasing motivation, adherence to treatment and strengthening the therapeutic alliance in multiple psychiatric disorders. In particular, patients with impulse control disorders and other disorders in which the patient suffers from inhibitory control deficits (e.g., behavioral addictions) have been shown to benefit from serious games.

**Aim**: The aim of this study was to describe the characteristics and to evaluate the usability of a new serious videogame, e-Estesia. This serious videogame was designed to improve emotion regulation in patients with gambling disorder (GD). Preliminary results from a pilot sample are also reported.

**Method**: A pilot sample of 26 patients undergoing treatment for GD was recruited (ranging from 22 to 74 years, mean = 41.2 and *SD* = 12.9; 80.8% men). Participants used e-Estesia on a tablet, which was connected to a thoracic band that sent heart rate (HR) and heart rate variability (HRV) data to the videogame platform in order to provide biofeedback. The System Usability Scale was completed by patients to determine the usability of e-Estesia.

**Results and Discussion**: e-Estesia performed comparatively well for all the explored groups (i.e., sex, age, and online vs. offline gambling: mean usability score = 83.8, *SD* = 13.1). Around 84.6% of the patients endorsed that it was easy to use. Female patients with GD presented higher HRV during the use of the serious videogame compared to men.

## Introduction

Gambling disorder (GD) is persistent and recurrent problematic gambling behavior leading to clinically significant impairment or distress (American Psychiatric Association, 2013). The prevalence of GD has been estimated to range between 0.12 and 5.8% (Calado and Griffiths, 2016), and lifetime prevalence rates for GD have been estimated to be around 0.5% (Petry et al., 2005; Kessler et al., 2008).

Several factors have been associated with severity in GD: psychiatric comorbidity (Pilver et al., 2013; Parhami et al., 2014), early age of onset (Johansson et al., 2009; Jiménez-Murcia et al., 2016), criminal behavior (Grant and Potenza, 2007; Folino and Abait, 2009; Granero et al., 2014), lack of emotion regulation (ER; Williams et al., 2012; Elmas et al., 2017; Rogier and Velotti, 2018; Marchica et al., 2020), or specific personality traits such as, high impulsivity (Álvarez-Moya et al., 2007; MacLaren et al., 2011; Black et al., 2014). The strong association between impulsivity and problematic gambling behavior has been extensively studied (Kräplin et al., 2014; Del Prete et al., 2017) and this construct has been described as a risk factor for GD (Blaszczynski and Nower, 2002; Johansson et al., 2009; Álvarez-Moya et al., 2011; Schreiber et al., 2012).

Emotion regulation is the ability to experience and modulate emotions (Gross, 1998). ER allows people to access functional resources in stressful situations, and allows for the use of appropriate coping strategies (Greenberg et al., 2007). According to the theoretical framework developed by Gratz and Roemer (2004), difficulties in the ER can be divided into six dimensions: reactions of non-acceptance of one’s difficulties and the tendency to experience negative secondary emotions in response to the experience of negative emotions (non-acceptance); difficulty in adopting goal-oriented behaviors and performing a task in the presence of negative emotions (goals); difficulty in controlling impulses and behaviors in the presence of negative emotions (impulse); lack of emotional awareness and willingness to pay attention to your emotions (awareness); difficulty in developing effective emotional regulation strategies once emotions have arisen (strategies); and lack of qualitative understanding of the emotion experienced (clarity).

Gambling can be used as a dysfunctional strategy to alleviate negative emotional states (Shead and Hodgins, 2009; Lloyd et al., 2010). It can serve as a maladaptive mechanism for regulating positive (e.g., euphoria and empowerment) and negative emotions (e.g., stress, boredom, or sadness; Tice and Bratslavsky, 2000). Individuals who suffer from GD present greater ER impairments than individuals without this disorder, significantly less use of reappraisal as an adaptive emotional regulation strategy or a counterproductive use of this strategy, a greater lack of emotional clarity and a greater lack of emotional awareness (Williams et al., 2012; Ruiz de Lara et al., 2019; Marchica et al., 2020).

Different studies indicate that difficulties in ER are associated with greater severity in GD (Álvarez-Moya et al., 2010; Lobo et al., 2014; Suomi et al., 2014). GD is a heterogeneous condition, and it is possible to identify differentiated subtypes, which share similar risk factors, phenotypes and symptoms. The prevalent pathway model of GD (Blaszczynski and Nower, 2002) describes the existence of two subgroups characterized by significant emotional vulnerability and emotional dysregulation (emotionally vulnerable subtype and the antisocial impulsive subtype). This theoretical model has been confirmed by empirical studies, both in clinical samples and in non-treatment-seeking individuals with gambling problems (Milosevic and Ledgerwood, 2010; Moon et al., 2017; Kurilla, 2021).

There is no widespread agreement between which type of intervention is the most appropriate to improve ER, despite the fact that the role of this aspect in the development, maintenance, and treatment of psychopathology has been widely shown in the literature (Berking et al., 2008, 2011; Sheppes et al., 2015; Jauregui et al., 2016). Different approaches have been proposed to address this issue, the most frequent being variations of CBT (Ducharme et al., 2012; Fagundo et al., 2013, 2014; Radkovsky et al., 2014), dialectic behavioral therapy (Geddes et al., 2013; Wallace et al., 2014), and mindfulness-based interventions (McIntosh et al., 2016; Sancho et al., 2018). For example, Sancho et al. (2018) reported in a systematic review of the literature that the mindfulness-based intervention in addictions was successful for improving emotion dysregulation and mood, as well as others symptoms (dependence, craving, depression, anxiety, and perceived stress).

Several new treatment perspectives in ER therapy using computer-based ER training or serious video-games have emerged (Jon Hobbs and Yan, 2008; Jiménez-Murcia et al., 2009; Kazdin and Blase, 2011; Ducharme et al., 2012; Fernández-Aranda et al., 2012). Although therapies based on cognitive-behavioral approaches continue to be the “gold standard” of treatment for GD, the use of technology-based tools complements this therapeutic approach with promising results for different health and mental conditions (Kato, 2010; Fernández-Aranda et al., 2012; Tárrega et al., 2015; Charlier et al., 2016; Jander et al., 2016). Among them, serious games have gained traction and have begun to be used in a variety of complementary mental health treatments (Lau et al., 2017; Barnes and Prescott, 2018). In recent years, a systematic review of usability literature in the context of serious games found that health was the second most frequent domain for the use of such games only after learning. Within health, serious games have been utilized to train patients and professionals, to provide education, and for diagnostic purposes (Yáñez-Gómez et al., 2017; Barnes and Prescott, 2018).

In its broadest conceptualization, a serious game is a tailor-made videogame specifically designed to target pre-defined goals (Kato, 2010). In recent years, videogames have moved beyond being considered as mere tools for entertainment and are now used to deliver complex and rich narratives that can be used for therapeutic purposes. There is even evidence to suggest that some video games are capable of generating more benefits in ER than the serious game specifically designed for this purpose, because the fictional properties that can be achieved are better, as they do not have to focus on another goal beyond the fun of the user (Villani et al., 2018). Among the frequently mentioned positive outcomes of playing videogames are the development of cognitive and social abilities, the inducement of positive emotions, improvements in mood, the promotion of engagement, and the achievement of self-actualizing experiences (Fernandez-Aranda et al., 2015; Tárrega et al., 2015; Osmanovic and Pecchioni, 2016; Mack et al., 2017; Russoniello et al., 2009).

The proliferation of serious games in health-related contexts has been explained as the consequence of three factors. First, serious games appeal to a wide audience that otherwise would not seek treatment; second, it is easier to engage in and enjoy the therapeutic process; and third, the immersive experiences on a sensory level (at the visual, auditory level, etc.) depicted in serious games have been shown to improve effectiveness (Fleming et al., 2017). Also, as self-training methods, these therapeutic aids can be easily accessible and affordable for those who face financial barriers in obtaining professional help for mental disorders (Clarke, 2007). A review of 15 studies reporting uses of serious games in psychotherapy concluded that all interventions showed positive results with improvements in the outcome variable, and were particularly effective in increasing motivation, adherence to treatment and strengthening the therapeutic alliance (Eichenberg and Schott, 2017). This final point is important, since the relationship between the therapeutic alliance and the response to treatment, regardless of the specific treatment modality used, has been demonstrated. Characteristics such as sex, age, or the type of problem presented by the patient influence the therapeutic alliance, in addition to the characteristics of the therapist and the interaction with the patient themselves (Martin et al., 2000; De Weert-Van Oene et al., 2001; Norcross, 2002; Arnow et al., 2013; Bucci et al., 2016; Kallergis, 2019). Another study by Tárrega et al. (2015) found that obtained patients had a lower dropout rate when using serious games compared to patients who did not.

Self-training of people experiencing GD *via* serious games with biofeedback sensors has been proven to reduce arousal and general impulsive behaviors while enhancing self-control (Jiménez-Murcia et al., 2009; Fernandez-Aranda et al., 2015; Tárrega et al., 2015). In the specific context of the use of serious games to channel ER interventions, some experiments have shown that participants prefer these over paper-based interventions, and that they find serious games more apt to generate, first frustration and then effective ER (Rodriguez et al., 2015).

The underlying mechanism of serious games for therapy is simple. Individuals become more aware of their emotions as measured by biosensors through a visual and audio representation of them on screen. The biofeedback connects the emotional reaction to the media display, helping individuals to learn how to regulate their emotions and visually rewarding them when doing adequately (Jerčić and Sundstedt, 2019). Serious games allow real situations to be recreated in virtual settings, while provoking a series of cognitive, emotional and behavioral responses in individuals. This allows for the training of specific skills in a motivating and entertaining way that can be more difficult to achieve with traditional therapies. For example, in the concrete task of uniting stars from the constellations in Playmancer, the core objective is training in relaxation, increased self-awareness and self-regulation. (Fernández-Aranda et al., 2012).

Bespoke games using biosignals to improve ER have been tested in laboratory conditions. One study reported that individuals increased their emotional awareness and improved their decision-making by refining reward processing through the biofeedback mechanism of the game (Astor et al., 2013). Heart rate variability (HRV) is an accessible research tool that can increase the understanding of emotion in social and psychopathological processes (Porges, 1986, 1997, 2001). HRV analysis is emerging as an objective measure of regulated emotional responding (generating emotional responses of appropriate timing and magnitude). The measurement of HRV responds to autonomic flexibility and, therefore, an increased HRV correlates with greater emotional control (Appelhans and Luecken, 2006).

In an experiment using a breathing-strategy-game for ER in youths, in which a frustration-inducing phase was added, participants did not behave differently when using mobile devices as compared to using a computer, signaling the adequacy of tablets for this purpose (Vara et al., 2016). However, that experiment was carried out among high school students and caution must be exerted to generalize these conclusions into a treatment-seeking setting. In fact, a systematic review revealed that 87% of health-oriented serious games usability studies were conducted on healthy users, whereas only 13% recruited a clinical sample (Yáñez-Gómez et al., 2017).

Since available empirical evidence in the scientific literature has indicated that sex and age play an important moderating role in gambling (Valero-Solís et al., 2018; Granero et al., 2020; Jiménez-Murcia et al., 2020), these two sociodemographic variables were considered.

Here, we present a new serious videogame, *e-Estesia*. Thus, the aims of this study are to first present a description of the serious videogame e-Estesia and then to assess its usability in patients undergoing treatment for GD. Differences in the usability scores based on the participants’ gender and age, as well as on gambling preferences, are also explored due the strong contribution of these features to gambling behavior.

## Materials and Methods

### Introducing e-Estesia

e-Estesia is an app-based serious videogame designed to improve ER in patients with impulse control disorders and other disorders characterized by inhibitory control deficits. In its current format, e-Estesia runs on password-protected Android portable devices (tablets) and is connected *via* Bluetooth to a sensor that transmits physiological data [heart rate (HR) and HRV] from the participant to the device.

The game is based on a previous serious game, developed by our group and the *PlayMancer* Consortium (Jiménez-Murcia et al., 2009; Fernández-Aranda et al., 2012). *PlayMancer* is a more complex serious videogame that retrieves scores from multiple biofeedback sensors including galvanic skin response, oxygen saturation, HR and HRV, breathing frequency and skin temperature. This intervention has been demonstrated to improve treatment outcomes in eating and GD patients (Fagundo et al., 2013; Fernandez-Aranda et al., 2015; Tárrega et al., 2015). However, *PlayMancer* needed patients to play the game within the premises of the hospital due to the multiple required biosensors. In contrast, e-Estesia is a much more convenient solution that facilitates treatment by (a) transforming the desktop computer-based experience into an app-based interface, making it fully portable and allowing treatment delivery at home, (b) reducing the outcome measures and the equipment needed to a single biosensor and a tablet, and (c) streamlining the game design and the human-media interactions to its core, most therapy-relevant components.

e-Estesia starts by asking participants about their mood. The question can be answered by selecting one of five face icons ranging from sadness to happiness (graphics validated in previous studies and in different samples; (Diana et al., 2012; Garcia-Palacios et al., 2014).

**Figure 1:**
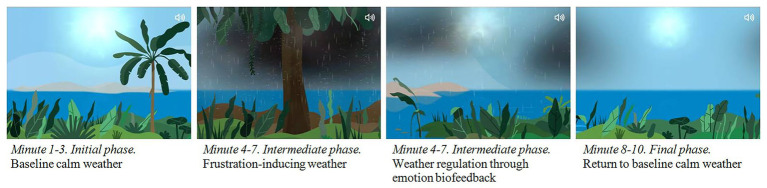
Screenshots from e-Estesia depicting the different phases of the Serious Game.

Next, a voice-over trains participants on how to become aware of their breathing and sitting posture. The game is set on a tropical island. The gamer’s point of view is located a few feet inland and moves alongside the coastline in a single, uninterrupted tracking shot (see Figure 1). The game depicts tropical vegetation, sea, horizon, sun, clouds, and rain. The game follows an A-B-A design pattern consisting of (a) an initial 3-min period in which the landscape is sunny, (b) a subsequent 4-min period in which rain clouds and eventually a storm appear, and (c) a final 3-min period identical to the first one. Gamers are instructed at the beginning of the game to try and breathe calmly (diaphragmatic breathing instructions).

The initial and final periods are used as baseline to practice this breathing technique. The intermediate period is designed for the subjects to learn how to manage their breathing through a HRV biofeedback mechanism. This mechanism gives the user information about their breathing thanks to a visual system with animations (e.g., clear sky vs. rainy clouds). In this way, e-Estesia reports feedback (clouds appear more or less frequently) based on participants’ breathing while using the device. When participants succeed in regulating their emotions by breathing properly, the biosensor utilizes this physiological information to reinforce this behavior by dissipating the storm; if breathing does not improve, the rain becomes more intense. This intermediate period adds stressors. During the game, simple instructions are given regarding how to modulate poor weather conditions. The patient must be an active participant in the process as they interact with the application and consolidate this learning *via* breathing techniques.

### Participants

A pilot sample of 26 patients seeking treatment for GD was obtained for the present study. Data from participants were collected from December 2018 to May 2019 by means of a purposive sample recruitment procedure. Consecutive patients who began cognitive-behavioral treatment for GD were offered the opportunity to participate in the study, and all participants who agreed were selected and provided that they met the inclusion and exclusion criteria. Due to the observational nature of this pilot study, self-selection bias and lack of randomization were not deemed significant as the results at this stage were not to be compared to a control group.

In order to participate, the following exclusion criteria were considered: (a) the presence of a psychiatric or neurological disorder such as schizophrenia or other psychotic disorders that might impact game performance, (b) an intellectual disability, and (c) active pharmacological therapy that might interfere with game performance. Inclusion criteria included being diagnosed with GD, which was assessed prior to the study both *via* screening methods (SOGS, DSM-IV criteria and other relevant measures to identify psychopathological symptoms) and individual structured interviews by experienced psychologists. The sample included 21 men (80.8%) and five women (19.2%), with ages ranging from 22 to 74 years (*M* = 41.2, *SD* = 12.9).

All participants received information regarding the aims of the research and they provided signed informed consent for participating. There was no financial or other compensation for being part of the study sample. Participants who agreed to take part in the study were briefed on the purpose of the study and were reassured of the voluntary nature of their participation and their rights to stop at any time. The study was approved by the Ethics Committee of the first author’s hospital (ref. number PR286/14), adhering to the principles outlined in the latest version of the Declaration of Helsinki.

### Procedure

After providing informed and signed consent, participants were scheduled for an individual appointment in a week’s time. This appointment was used to (a) brief the participants about the study procedure; (b) explain how to use the device and sensor strap; (c) train participants how to “beat” the app (i.e., breathing calmly, regulating one’s emotions, etc.), and (d) conduct an initial supervised session using e-Estesia. After conducting the initial intervention session with the application, the usability scale described below was administered to the participants.

### Measures

The *System Usability Scale* (SUS; Brooke, 1996) was used to gauge how usable participants perceived the app to be. This scale is context-specific and does not measure usability in isolation but as a product of the user-interface interaction and the specific goals that users expect to accomplish with its use (Schmidt and De Marchi, 2017). The instrument was conceived after the international standard ISO 9241-11, which identifies effectiveness, efficiency, and satisfaction as the key components of usability assessment. The instrument comprises 10 items and each item is placed on a 5-point Likert scale (from 1 to 5; 1 = strongly disagree; 3 = neither agree nor disagree; 5 = in complete agreement). Final scores for SUS range from 0 to 100, with higher scores indicating greater usability. Internal consistency of the scale calculated in the study was adequate (Cronbach-alpha, *α* = 0.75).

### Psychophysiological Variables

Two physiological variables were measured: heart rate (HR) and HRV (defined as the variation in the time between successive heartbeats). These measurements were recorded *via* a *Polar H7 Bluetooth Heart Rate Sensor* by Polar Electro Oy^TM^. HR (beats per minute) scores were used. The rationale for these measures derives from previous studies pointing out that deviations higher than baseline HR scores are typically associated with emotions of anger and joy, whereas lower values of excitement (i.e., below baseline) usually indicate neutral condition and boredom (Eddie et al., 2014, 2015). HRV, which usually ranges from 30 to 39, is a variation in the beat-to-beat time interval (time between two successive R-waves) that occurs spontaneously and naturally and that is positively associated with self-regulation, heart health, autonomous balance, and task performance. In turn, it is negatively associated with anxiety, depression, stress, hypertension, chronic muscle pain, and nausea (Porges, 1995; Malik, 1996; Sargunaraj et al., 1996; Litchfield, 2003; Lehrer et al., 2010; Lehrer and Gevirtz, 2014; Russell et al., 2014; Nazarewicz et al., 2015).

### Sociodemographic Variables

Additional data were obtained through a semi-structured interview, which assessed sociodemographic features (e.g., education, marital status, socio-economic, and employment status). However, for the purpose of the present study, only age, gender, and gambling platform preference (i.e., offline vs. online) were reported.

### Statistical Procedure

Statistical analysis was done with Stata16 for Windows. Means on the SUS were compared between the groups defined by the patients’ sex and age, through nonparametric Mann-Whitney tests, and effect size for the mean differences was estimated through Cohen’s-*d* coefficient (|*d*| < 0.20 was considered null effect size, |*d*| < 0.50 low, |*d*| > 0.50 moderate and |*d*| > 0.80 large).

## Results

The sample included 21 men (80.8%) and five women (19.2%), with ages in the range of 22–74 years-old (*M* = 41.2, *SD* = 12.9). The number of participants who reported to prefer offline gambling was 21, whereas five preferred online gambling.

Table 1 includes the frequency distribution of the items in the study. Total raw score for the SUS ranged from 53 to 98 (mean = 83.8, *SD* = 13.1; Normality test: Kolmogorov-Smirnov-Statistic = 0.13, *df* = 26, *p* = 0.200). Table 2 contains the results of the comparison of mean scores based on the patients’ sex, gambling preferences, and age groups [defined by the median (percentile 50) in the sample]. Although no statistical differences were obtained in the comparison between the groups, a moderate effect size was related to participants’ age (lower mean score in the younger age group; |*d*| = 0.59).

**Table 1:** Frequency distribution for the System Usability Scale (SUS) items.

		Strongly disagree								Strongly agree	
		Option 1		Option 2		Option 3		Option 4		Option 5	
		*n*	%	*n*	%	*n*	%	*n*	%	*n*	%
1.	I would like to use this system	1	3.8%	3	11.5%	9	34.6%	8	30.8%	5	19.2%
2.	System unnecessarily complex	19	73.1%	1	3.8%	4	15.4%	1	3.8%	1	3.8%
3.	System easy to use	1	3.8%	0	0%	3	11.5%	4	15.4%	18	69.2%
4.	I would need technical support	21	80.8%	5	19.2%	0	0%	0	0%	0	0%
5.	Various functions well integrated	0	0%	3	11.5%	7	26.9%	9	34.6%	7	26.9%
6.	Too much inconsistency	11	42.3%	10	38.5%	4	15.4%	1	3.8%	0	0%
7.	Most people would learn	0	0%	1	3.8%	2	7.7%	6	23.1%	17	65.4%
8.	Very cumbersome to use	22	84.6%	1	3.8%	2	7.7%	0	0%	1	3.8%
9.	Very confident using the system	1	3.8%	2	7.7%	4	15.4%	8	30.8%	11	42.3%
10.	I need a lot of learn before use	22	84.6%	2	7.7%	1	3.8%	0	0%	1	3.8%

**Table 2:** Comparison of the SUS raw scores divided by sex, gambling preference and groups of age.

Sex						Gambling preference						Age groups					
Women *n* = 5		Men *n* = 21				Offline *n* = 21		Online *n* = 5				Age 22–35 *n* = 12		Age 36–74 *n* = 14			
*Mean*	*SD*	*Mean*	*SD*	*p*	*d*	*Mean*	*SD*	*Mean*	*SD*	*p*	*d*	*Mean*	*SD*	*Mean*	*SD*	*p*	*d*
86.5	8.8	81.4	13.9	0.447	0.44	81.8	11.7	85.0	19.2	0.631	0.20	78.3	13.7	85.9	11.9	0.145	**0.59**^†^

SD, standard deviation. Groups of age defined for the median (percentile 50) in the sample.

† Bold: effect size in the medium (|*d*| > 0.50) to large (|*d*| > 0.80) range.

**Figure 2:**
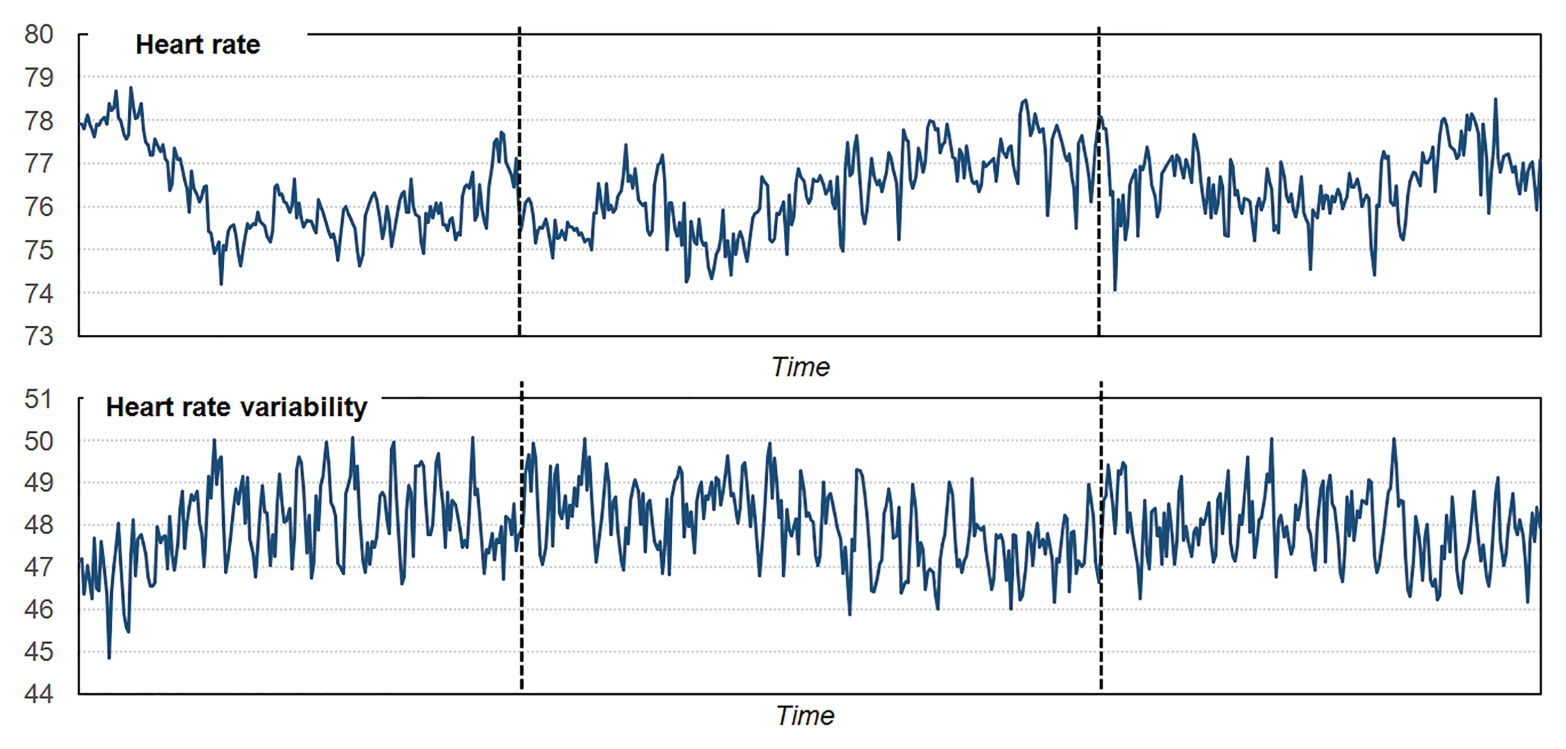
Line-plot for heart rate (HR) and heart rate variability (HRV) means within the entire sample.

**Figure 3:**
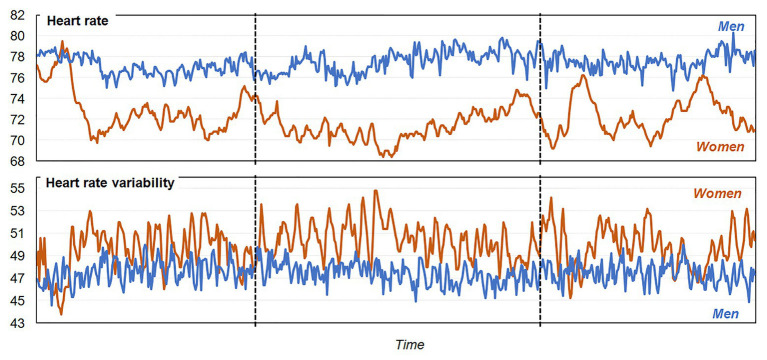
Line-plot for HR and HRV means stratified by sex.

Regarding the preliminary results of the pilot study carried out using e-Estesia, Figure 2 shows the changes in HR and HRV during the initial 3-min period pre-game, the subsequent 4-min period game play, and the 3-min period post-game, obtained in the sample (*n* = 26). As expected, the measures varied as a function of time in the session, with increased activity during the start of the game (differences are more evident for HR comparing the period of game play vs. the period of pre-game). Figure 3 includes the line-plot stratified by the patients’ sex, which shows greater activation in HR among men compared to women for all the session, but greater HRV among women. Particularly, women showed a decrease in the HR during the period of game play compared with the pre-game phase, while men showed an increase in the HR levels during the game playing phase.

## Discussion

The present study aimed to assess the usability of a new emotion-regulation training app for GD, based on previous studies carried out in eating disorders and GD (Fernández-Aranda et al., 2012). In the study participated 26 patients undergoing treatment for GD, giving high scores in effectiveness, efficiency, and satisfaction with the use of the tablet-based serious game e-Estesia (according to the SUS; Brooke, 1996).

This is one of the few studies conducted in order to evaluate usability of a serious game in clinical population (Yáñez-Gómez et al., 2017). The app performed comparatively well in all groups. The average score, both in men and women, and with different gambling preferences (online vs. offline) was over 80 (ranging from 0 to 100), which indicates a high levels of endorsed usability. Mean scores were somewhat lower in the younger group (between 22 and 35 years).

The main reasons for using this specific type of technology are the internal characteristics of serious games, such as the intensity with which they capture the attention of the individual, the ease with which they disconnect from the outside world, their ability to immerse, the low resistance on the part of most individuals to use them and the motivation that they tend to generate in many people (Eichenberg and Schott, 2017). With *e-Estesia*, aspects as the easy use, easy learning, and the no expert support was needed in it use were highly scored (84, 88, and 100% of agreement, respectively). This is an important aspect considered that one of the aims of the development of *e-Estesia* were to provide independence to their users, as an improvement of other similar serious games that have been used to improve emotional regulation, such as *Playmancer*, this serious game is useful for improving emotional regulation, but requires the support of an expert to be able to use it (Fernández-Aranda et al., 2012).

Some studies have shown that the use of video games could improve motivation and adherence to treatment (Kato et al., 2008; Coyle et al., 2009). The study results showed that a high percentage of participants believed the app was coherent, consistent, not confusing (61.5, 80, and 88.4% of agreement respectively) and that its features were adequately merged into the overall app design and content. This aspect is important considering that the benefits of the use of serious games in health contexts are related with their capacity to capture the attention of the user, which can help the motivation to continue using it (Fagundo et al., 2013). Also, in the context of GD patients, this could be an important auxiliary treatment strategy, if it is considered that different personality traits (Tárrega et al., 2015), such as low self-directedness (Janiri et al., 2007), have been associated with lack of adherence to the treatment (Del Pino-Gutiérrez et al., 2017).

Finally, comfort and desire to use the app were inextricably linked. All the patients who responded that they agreed that they would like to use the application frequently also agreed that they were comfortable using it. This is consistent with the preexisting literature that affirms that for serious game users, it is easier to engage in the therapeutic process if it is enjoyable (Fleming et al., 2017).

Using e-Estesia during a session with 26 GD patients revealed that women had a lower mean HR and a higher HRV during the use of the serious game compared to men. This could indicate that for this first session, women show a greater capacity to self-regulate their psychophysiological state through biofeedback and breathing instructions (Appelhans and Luecken, 2006). During the interaction phase of the serious game, this difference between men and women increased, with women showing a higher HRV.

These results should be careful lighted through the differences in emotional regulation difficulties that both genders present in GD patients. While higher rates of arousal-seeking behavior have been described in men, women’s gambling activities may be related with the aim of avoid negative emotion states (Ledgerwood and Petry, 2006; Liu et al., 2009), for which it is possible to hypothesized that men GD patients could have higher difficulties at the beginning in self-regulation of them psychophysiological state. However, other aspects as the conditions of the evaluation, may influenced these results. A study comprising 484 GD patients showed that men had more difficulties in ER compared to women (specifically, men had poorer performance in the non-acceptance of emotions domain; Sancho et al., 2019). Other studies in laboratory conditions and with healthy populations show results contrary to the study, suggesting that women could react to laboratory stressors with higher HR responses, whereas men have greater blood pressure (Carrillo et al., 2001; Abad-Tortosa et al., 2017).

This application aims to improve the management of ER through a biofeedback mechanism, mediated by instructions in diaphragmatic breathing. It is possible that in the first sessions, in which patients are learning the management of their psychophysiological state and the establishment of new breathing patterns, they would not feel entirely comfortable with this intervention. Levels of stress and anxiety might even be expected to increase during the first sessions. Other serious game studies have shown similar results on this point, such as the “Zen Cat,” a brain-computer interface that allow any person to control applications using only their brain waves. The majority of players, in the first sessions of the evaluation, thought that the game was hard, even on the easy mode. Although this opinion persisted for some subjects in the later sessions as they were still trying to find a good strategy, most of them thought that the game became easier with each session, as they were able to reach higher scores and maintain the average meditation higher for longer periods of time.

Therefore, future research is needed that measures more than one e-Estesia session in contexts that differ from the laboratory. In conclusion, the usability scores and the biofeedback information provide promising evidence of using e-Estesia to assist in the treatment of GD.

## Limitations and Future Lines of Research

This study should be interpreted in the basis on some limitations. First, the usability of the app was assessed through a scale focused on the key components of this type of systems, and, therefore, no reference benchmarks or norms are available for comparing our results. In addition, only one scale was used to measure usability, without specific additional methods such as usability testing, cognitive walkthrough, heuristics analysis or other instruments. Second, the low sample size: statistical analysis was not adequately powered to examine associations between background variables with the usability measures. It should be outlined that many feasibility studies are carried out within small samples, which should accurately reflect the characteristics of the larger target population of users. We tried to choose a representative group of GD patients that closely matched the distribution of age, sex, and gambling preference of the whole population candidate to use the e-Estesia. In Spain, the odds men/women and offline/online preference among GD patients are around 4/1, and these ratios have been reflected in this pilot analysis. Future studies could test additional relationships, and try to identify potential predictors of usability in the GD area (such as the presence of comorbid mental health conditions, sociodemographic profile or confidence in using technology).

## Data Availability Statement

The raw data supporting the conclusions of this article will be made available by the authors, without undue reservation.

## Ethics Statement

The studies involving human participants were reviewed and approved by Ethics Committee of the first author’s hospital (ref. number PR286/14), adhering to the principles outlined in the latest version of the Declaration of Helsinki. The patients/participants provided their written informed consent to participate in this study.

## Author Contributions

SJ-M, FF-A, TM-M, RG, LM, and TS contributed to the development of the study concept and design. SJ-M, FF-A, RG, LM, AP-G, ML-M, MG-P, LM, and JM had previous experience in the design and use of SG for the treatment of impulsive spectrum disorders and their experience and advice were decisive in the design of the present device. RG performed the statistical analysis, wrote the results and made the tables and Figures. SJ-M, TM-M, LM, TS, and HL-G aided with interpretation of data and the writing of the manuscript. TM-M, LM, AP-G, ML-M, BM-M, EV-M, MG-P, and LM collected the data. FF-A, JM, IG, TS, MG-B, and AS revised the manuscript and provided substantial comments. SJ-M, FF-A and JM obtained funding. All authors contributed to the article and approved the submitted version.

### Conflict of Interest

The authors declare that the research was conducted in the absence of any commercial or financial relationships that could be construed as a potential conflict of interest.
